# Human Papillomavirus-Related Cancers Among People Living With AIDS in Puerto Rico

**DOI:** 10.5888/pcd11.130361

**Published:** 2014-05-15

**Authors:** Ana Patricia Ortiz, Javier Pérez-Irizarry, Marievelisse Soto-Salgado, Erick Suárez, Naydi Pérez, Maritza Cruz, Joel Palefsky, Guillermo Tortolero-Luna, Sandra Miranda, Vivian Colón-López

**Affiliations:** Author Affiliations: Javier Pérez-Irizarry, Naydi Pérez, Guillermo Tortolero-Luna, University of Puerto Rico Comprehensive Cancer Center, San Juan, Puerto Rico; Marievelisse Soto-Salgado, Erick Suárez, Vivian Colón-López, University of Puerto Rico, San Juan, Puerto Rico; Maritza Cruz, Sandra Miranda, Puerto Rico Department of Health, HIV Surveillance Program, San Juan, Puerto Rico; Joel Palefsky, University of California, San Francisco, San Francisco, California.

## Abstract

The objective of this study was to estimate the incidence of cancer and human papillomavirus (HPV)–related cancers and the risk of death (by cancer status) among people living with AIDS (PLWA) in Puerto Rico. We used data from the Puerto Rico AIDS Surveillance Program and Central Cancer Registry (1985–2005). Cancers with highest incidence were cervix (299.6/100,000) for women and oral cavity/oropharynx for men (150.0/100,000); the greatest excess of cancer incidence for men (standardized incidence ratio, 86.8) and women (standardized incidence ratio, 52.8) was for anal cancer. PLWA who developed a cancer had decreased survival and increased risk of death compared with those who did not have cancer. Cancer control strategies for PLWA will be essential for improving their disease survival.

## Objective

Human papilloma virus (HPV) infections and HPV-related cancers are more common in people living with AIDS (PLWA) than in the general population (1,2). Although the incidence of cancer has diminished with the advent of highly active antiretroviral therapy (HAART), it has not diminished for certain HPV-related cancers (1–4). HPV-related malignancies have a distinct etiology, characterized by epithelial damage induced by persistent infection (1). Puerto Rico has a high burden of HPV-related cancers and HIV/AIDS (5,6). The objective of this study was to estimate the incidence of cancer and HPV-related cancers and the risk of death (by cancer status) among PLWA in Puerto Rico and compare these statistics with those in the general Puerto Rican population.

## Methods

This study was approved by the institutional review board of the University of Puerto Rico Medical Sciences Campus in October 2010. We linked data from the Puerto Rico AIDS Surveillance Program and the Puerto Rico Central Cancer Registry by using Link Plus version 2.0 software (Centers for Disease Control and Prevention, Atlanta, Georgia) to describe the cancer profile of PLWA (aged ≥15 y) who were diagnosed with cancer from January 1, 1985, through December 31, 2005. We limited our study to invasive primary cancers diagnosed 3 months after an AIDS diagnosis (6). Overall, 29,806 cases met our inclusion criteria; we established 3 categories of cancer status: no cancer (n = 29,065), non-HPV–related cancer (n = 672), and HPV-related cancer (n = 69).

We included the following HPV-related cancers: cancers of the cervix, vulva/vagina, penis, anus, and oral cavity/oropharynx (1,5); a subanalysis considered only HPV-related histology (7). We grouped cases according to period of AIDS diagnosis: 1985–1995 (Pre-HAART) and 1996–2005 (HAART). Using χ^2^ tests, we compared the demographics of the study population by cancer status. The follow-up period of cancers among PLWA was until the date of death or December 31, 2008 (whichever occurred first). For the cancer risk analysis, we considered first and subsequent malignancies. The standardized incidence ratio (SIR) was estimated by using the indirect method and was defined as the observed cancer incidence divided by the expected cancer incidence based on Puerto Rico population rates (2000–2004) (8). SIR values were estimated by period of AIDS diagnosis, sex, and cancer status. We also measured the median survival time of PLWA to describe survival by cancer status and period of AIDS diagnosis. To assess the risk of death we estimated the hazard ratio (HR) of death with 95% confidence intervals (CIs) by using the Cox proportional hazards model, stratified by sex and period of AIDS diagnosis. Cases lost to follow-up and those alive at December 31, 2008, were censored. The proportional hazards assumption of the Cox model was tested and validated and an interaction assessment was performed. We used Stata 12.0 (Stata Corp, College Station, Texas) for the statistical analysis.

## Results

The distribution of PLWA varied by sex, age, mode of HIV exposure, and period of AIDS diagnosis (Table 1). The proportion of women who had an HPV-related cancer was larger than the proportion of women who had a non-HPV–related cancer or no cancer; we found similar results for PLWA whose HIV was transmitted heterosexually.

**Table 1:** Demographic Characteristics of People Living With AIDS in Puerto Rico (N = 29,806), by Cancer Status, 1985–2005^a^

Characteristic	1985–1995				1996–2005			
	No Cancer	Non-HPV–Related Cancer	HPV-Related Cancer	*P* Value^b^	No Cancer	Non-HPV–Related Cancer	HPV-Related Cancer	*P* Value^b^
**Total, n**	16,858	447	34	NA	12,207	225	35	NA
**Sex**								
Male	13,274 (78.7)	393 (87.9)	23 (67.6)	<.001	8,830 (72.3)	181 (80.4)	17 (48.6)	<.001
Female	3,584 (21.3)	54 (12.1)	11 (32.4)		3,377 (27.7)	44 (19.6)	18 (51.4)	
**Age at AIDS diagnosis, y**								
15–29	3,575 (21.2)	103 (23.0)	6 (17.6)	.74	1,507 (12.4)	21 (9.3)	5 (14.3)	.003
30–39	7,806 (46.3)	203 (45.4)	15 (44.1)		4,773 (39.1)	71 (31.6)	16 (45.7)	
40–49	3,859 (22.9)	98 (21.9)	7 (20.6)		3,870 (31.7)	72 (32.0)	10 (28.6)	
≥50	1,618 (9.6)	43 (9.6)	6 (17.6)		2,057 (16.8)	61 (27.1)	4 (11.4)	
Median, y	35	36	32	.10^c^	39	43	40	.02^c^
**Mode of HIV exposure**								
MSM	2,567 (15.2)	173 (38.7)	9 (26.5)	<.001	1,898 (15.6)	57 (25.3)	4 (11.4)	<.001
IDU	9,261 (54.9)	151 (33.8)	13 (38.2)		5,508 (45.1)	63 (28.0)	11 (31.4)	
MSM and IDU	1,401 (8.3)	44 (9.8)	1 (2.9)		682 (5.6)	21 (9.3)	2 (5.7)	
Heterosexual	3,283 (19.5)	75 (16.8)	10 (29.4)		3,962 (32.5)	81 (36.0)	18 (51.4)	
Other/unknown	346 (2.0)	4 (0.9)	1 (2.9)		157 (1.3)	3 (1.3)	0	

Abbreviations: HPV, human papillomavirus; NA, not applicable; MSM, men who have sex with men; IDU, injection drug use.

a The study population included people living with AIDS (aged ≥15 y) who were diagnosed with cancer from January 1, 1985, through December 31, 2005, 3 months after an AIDS diagnosis. Data for people who did not meet inclusion criteria for the study were not included in this table. All values are number (percentage) unless otherwise indicated.

b χ^2^ test, except for median age.

c One-way analysis of variance.

The highest incidences were for cervical cancer (299.6/100,000) among women and for oral cavity/oropharyngeal cancers (150.0/100,000) among men; anal cancer was the second leading cancer among both sexes. We found an excess of cancer incidence (overall, HPV-related, and non-HPV–related) among PLWA during both periods of AIDS diagnosis. Among HPV-related cancers, the greatest excess of incidence was for anal cancer among men (SIR = 86.8; 95% CI, 51.5–137.2) and women (SIR = 52.8; 95% CI, 10.9–154.3). We observed similar patterns in both time periods and for certain HPV-related histologies (Table 2).

**Table 2:** Incidence and Standardized Incidence Ratio (SIR)^a^ of Cancer Among People Living With AIDS in Puerto Rico, 1985–2005

Cancer type	1985–1995		1996–2005		1985–2005		1985–2005 (Based on HPV-Related Histologies^b^)	
	Incidence (per 100,000)	SIR (95% CI)	Incidence (per 100,000)	SIR (95% CI)	Incidence (per 100,000)	SIR (95% CI)	Incidence (per 100,000)	SIR (95% CI)
Overall	5,907.4	16.3 (14.9–17.8)	3142.0	8.7 (7.7–9.7)	4,501.9	12.4 (11.5–13.3)	4,512.8	12.3 (11.5–13.3)
Non-HPV–related	5,613.9	17.2 (15.6–18.8)	2750.7	8.4 (7.4–9.6)	4,153.9	12.7 (11.7–13.7)	4,293.2	12.4 (11.5–13.3)
HPV-related	304.9	13.0 (9.0–18.2)	350.6	15.0 (10.5–20.6)	327.1	13.8 (10.8–17.6)	228.9	11.8 (8.8–15.6)
**Women**								
Cervix	220.5	18.2 (7.9–35.9)	370.5	30.7 (17.1–50.5)	299.6	24.7 (15.7–37.1)	66.5	5.8 (3.7–8.8)
Vulva/vagina	79.1	22.6 (0.6–126.0)	52.8	15.1 (0.38–84.2)	63.3	18.2 (2.2–65.4)	—	—
Oral cavity/oro-pharynx	67.4	12.5 (0.32–69.9)	47.8	8.9 (0.22–49.6)	55.9	10.4(1.26–37.6)	—	—
Anus	91.8	47.7 (1.2–265.6)	107.4	55.8 (6.8–201.5)	101.7	52.8 (10.9–154.3)	98.8	62.7 (12.9–183.1)
**Men**								
Penis	0	0 (0–17.3)	29.9	9.7 (1.2–35.1)	14.7	4.8 (0.6–17.3)	11.3	4.0 (0.5–14.4)
Oral cavity/oro-pharynx	156.1	7.9 (3.9–14.0)	143.9	7.2 (3.47–13.3)	150.0	7.6 (4.7–11.6)	50.5	3.1 (1.3–6.5)
Anus	124.0	107.2 (55.4–187.3)	72.7	62.3 (23.1–136.9)	100.4	86.8 (51.5–137.2)	74.4	125.8 (71.9–204.4)

Abbreviations: HPV, human papillomavirus; CI, confidence interval; —, could not be calculated because of small numbers.

a The expected cases for SIRs were estimated by using the Puerto Rico Incidence Cancer File (8).

b Case definitions based on expert consensus were used to recalculate the burden of HPV-related invasive cancers at anatomic sites (cervix, vulva/vagina, penis, anus, and oral cavity and oropharynx) and for cell types (carcinoma of the cervix [ICD-O-3 histology codes 8010–8671 and 8940–8941] and squamous [ICD-O-3 histology codes 8050–8084 and 8120–8131] cells for other sites) in which HPV DNA is frequently found (1). This definition resulted in the reclassification of only 10 malignancies to the non-HPV–related cancer category (59 HPV-related cancers, 682 non-HPV–related cancers).

Overall, the median follow-up time varied by cancer status and period of AIDS diagnosis; we found longer survival times during 1996–2005 and among PLWA with no cancer (1985–1995, 2.1 y; 1996–2005, 7.5 y) than those with an HPV-related cancer (1985–1995, 0.8 y; 1996–2005, 2.6 y) or a non-HPV–related cancer (1985–1995, 0.6 y; 1996–2005, 0.7 y) (Wilcoxon *P* <.001). Cox models (HR [95% CI]) adjusted by age at AIDS diagnosis showed that among men and women, those diagnosed with a non-HPV–related cancer had a higher risk of death than those with no cancer:


**Sex**

**1985–1995**

**1996–2005**

**Men**
1.40 (1.24–1.58)1.95 (1.61–2.36)
**Women**
1.85 (1.32–2.61)2.31 (1.59–3.35)

Although no excess risk of death was observed for women with HPV-related cancers compared with those who had no cancer, men diagnosed with these cancers had a higher risk of death than those who had no cancer (HR [1985–1995] = 1.27; 95% CI, 0.81–2.00 and HR [1996–2005] = 1.32; 95% CI, 0.71–2.46); however, these risk excesses were not significant (*P* > .05).

## Discussion

Our study updates information on the cancer burden among PLWA in Puerto Rico with a focus on HPV-related cancers and presents the first statistics on cancer survival and risk of death for this group. Consistent with studies worldwide and in Puerto Rico (6), the burden of cancer (9) and HPV-related cancers (1,2,10,11) was higher among PLWA than among the general population. Although comparisons should be made cautiously because of the different methods used by these studies, our study suggests higher excess incidence of cancer and HPV-related cancers among PLWA in Puerto Rico than in other populations (2,9,11). 

In both periods of diagnosis, the highest excess incidence for cancer was for anal cancer. This result highlights the need for anal cancer screening among PLWA, although further research on this area is warranted (12,13). Given the lack of guidelines on anal cancer screening, clinical trials that determine the effectiveness of the Papanicolaou test for anal cancer prevention are needed (11). HPV vaccination (4) should be promoted in Puerto Rico, where vaccine uptake is low (14). Young PLWA should be targeted in vaccination efforts, although additional studies of vaccine efficacy among PLWA are needed (15).

We also documented decreased survival and increased risk of death (significant only for non-HPV–related cancers) among PLWA who developed a cancer compared with those who did not. Our study supports the importance of strengthening cancer screening and providing access to care among PLWA to decrease the incidence of cancer and improve survival and quality of life. Although the small number of HPV-related cancers among PLWA reduces the precision of our estimations, we conclude that PLWA in Puerto Rico have a greater burden of cancer than the general population, and this burden has a negative impact on survival. Further research and cancer prevention and control strategies are needed to reduce health disparities among PLWA in Puerto Rico. The cancer and HIV/AIDS surveillance systems should collaborate in cancer surveillance among PLWA for disease monitoring and intervention assessment.
